# ProNGF Expression and Targeting in Glioblastoma Multiforme

**DOI:** 10.3390/ijms24021616

**Published:** 2023-01-13

**Authors:** Mark Marsland, Amiee Dowdell, Sam Faulkner, Phillip Jobling, Robert A. Rush, Craig Gedye, James Lynam, Cassandra P. Griffin, Mark Baker, Joanne Marsland, Chen Chen Jiang, Hubert Hondermarck

**Affiliations:** 1 School of Biomedical Sciences and Pharmacy, College of Health, Medicine and Wellbeing, University of Newcastle, Callaghan, NSW 2308, Australia; 2 Hunter Medical Research Institute, University of Newcastle, New Lambton Heights, NSW 2305, Australia; 3 Biosensis Pty Ltd., Thebarton, SA 5031, Australia; 4 School of Medicine and Public Health, College of Health, Medicine and Wellbeing, University of Newcastle, Callaghan, NSW 2308, Australia; 5 Department of Medical Oncology, Calvary Mater, Newcastle, NSW 2298, Australia; 6 Hunter Cancer Biobank, NSW Regional Biospecimen and Research Services, University of Newcastle, Callaghan, NSW 2305, Australia

**Keywords:** glioblastoma multiforme, proNGF, biomarker, therapeutic target

## Abstract

Glioblastoma multiforme (GBM) is the most lethal adult brain cancer. Temozolomide (TMZ), the standard chemotherapeutic drug used in GBM, has limited benefit and alternate therapies are needed to improve GBM treatment. Nerve growth factor (NGF) and its precursor proNGF are increasingly recognized as stimulators of human tumor progression. The expression and stimulatory effect of NGF on GBM cell growth has previously been reported, but the status of proNGF in GBM is unreported. In this study, we have investigated proNGF expression and biological activity in GBM. A clinical cohort of GBM (*n* = 72) and low-grade glioma (*n* = 20) was analyzed by immunohistochemistry for proNGF and digital quantification. ProNGF expression was significantly increased in GBM compared to low grade gliomas and proNGF was also detected in patient plasma samples. ProNGF was also detected in most GBM cell lines by Western blotting. Although anti-proNGF blocking antibodies inhibited cell growth in GBM cells with methylated MGMT gene promoter, targeting proNGF could not potentiate the efficacy of TMZ. In subcutaneous xenograft of human GBM cells, anti-proNGF antibodies slightly reduced tumor volume but had no impact on TMZ efficacy. In conclusion, this data reveals that proNGF is overexpressed in GBM and can stimulate cancer cell growth. The potential of proNGF as a clinical biomarker and therapeutic target warrants further investigations.

## 1. Introduction

Glioblastoma multiforme (GBM) is the most aggressive brain cancer in adults. GBM accounts for 45% of malignant primary brain tumors. Median overall survival is often quoted at around 15 months, but this is true only for the fraction of people well enough for clinical trials; survival is often less than one year in community series [1]. Temozolomide (TMZ) is the oral alkylating agent used as first-line chemotherapy [2]. However, resistance to TMZ invariably occurs, particularly in the case of O6-methylguanine-DNA methyl transferase (MGMT) gene promoter methylation [3] and only around 7% of patients with GBM survive 5 years or longer [4]. New therapeutic targets and innovative therapies need to be identified for GBM to improve the efficacy of TMZ and patient outcomes.

Nerve growth factor (NGF) and its receptors the tyrosine kinase NTRK1 (also called TrkA) and the neurotrophin receptor p75^NTR^ (also called CD271) are expressed in many human malignancies and are increasingly reported as therapeutic targets in oncology [5]. NGF is expressed in GBM and has been shown to stimulate GBM cell proliferation through a mechanism involving the stimulation of both NTRK1 [6,7,8] and p75^NTR^ [9,10]. NGF is initially produced as a biochemical precursor (proNGF), a soluble 246 amino acid pro-peptide transcribed from the nerve growth factor (NGF) gene. ProNGF can subsequently be cleaved into NGF, either intracellularly or extracellularly after secretion, by tissue proconvertases such as furin and matrix metalloproteinases, respectively [11]. Importantly, proNGF is not only a metabolic precursor of NGF, but elicits its own biological activity by promoting neuronal differentiation or apoptosis, depending on the relative expression of its receptors p75^NTR^ and sortilin [12]. Interestingly, proNGF has been associated with progression and aggressiveness of several malignancies, including breast cancer [13,14], prostate cancer [15] and melanoma [16]. In thyroid cancer, proNGF is increased [17] and proNGF levels are higher in thyroid cancer lymph node metastases than in primary tumors but is not associated with high-risk clinical features [18,19]. While proNGF has previously been reported to be secreted from the GBM cell line U87MG [20], the expression and potential function of proNGF in patient samples is unclear.

In the present study, we have investigated the expression and biological effect of proNGF in GBM patient specimens and preclinical models, respectively. ProNGF was found to be overexpressed in GBM tumors and detectable in the blood of some GBM patients. Blocking anti-proNGF antibodies inhibited GBM cell growth both in vitro and in vivo, however, they could not potentiate the efficacy of TMZ. These data highlight that proNGF may participate in GBM progression and further preclinical and clinical investigations are warranted to define its potential value as a clinical biomarker and therapeutic target.

## 2. Results

### 2.1. ProNGF Expression in GBM Tissues

**Figure 1:**
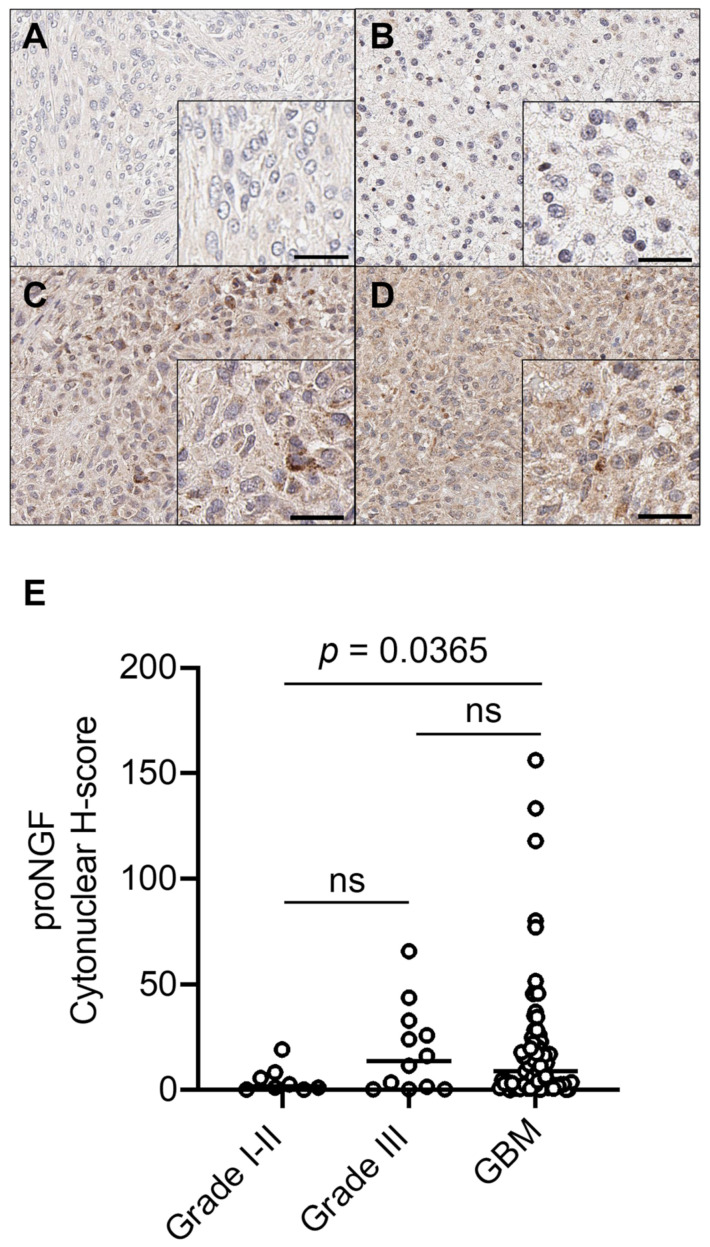
ProNGF expression in GBM vs. low grade gliomas. Immunohistochemical detection of proNGF, representative pictures are shown for (**A**) grade I, (**B**) grade II, (**C**) grade III gliomas, and (**D**) GBM. (**E**) Digital quantification of proNGF staining intensities according to grouped pathological subtypes: grade I–II (h-score = 1.98, IQR 0.50–7.73), grade III (h-score = 13.93, IQR 0.74–31.15) and GBM (h-score = 9.05, IQR 2.63–22.31). Scale bar = 30 µm. Data are expressed as individual values with medians. H-score distributions were compared using the Wilcoxon rank-sum (dichotomous) or Kruskal–Wallis (multiple comparisons) tests. Mann-Whitney test was used to evaluate the proNGF h-score median difference between grades I–II and GBM, and grade III and GBM.

ProNGF expression in GBM tissues is reported in Figure 1 and Table 1. ProNGF was observed in grade I gliomas at low and high staining intensities (Figure 1A). In all grade II cases, proNGF IHC staining was observed to be at a low intensity (Figure 1B). Grade III showed high staining intensity in seven of twelve cases (Figure 1C). ProNGF IHC staining in GBM was observed to have the highest staining intensity (Figure 1D). Digital quantification of proNGF protein expression (Figure 1E) confirmed a higher proNGF staining intensity in GBM (median h-score = 9.05, IQR 2.63–22.31) compared to grade I–II (low grade glioma) (median h-score = 1.98, IQR 0.50–7.73, *p* = 0.0365). Table 1 presents the association between clinicopathological parameters in GBM and proNGF intensity staining.

Immunohistochemical staining in each tissue sample was digitally quantified and categorized as low staining (≤median h-score), or high staining (>median h-score). A Chi-squared test was used to test statistical association. Statistically significant *p* values (*p* < 0.05) are shown in bold. ProNGF is the precursor for nerve growth factor.

### 2.2. Quantification of proNGF in Plasma from GBM Patients

**Figure 2:**
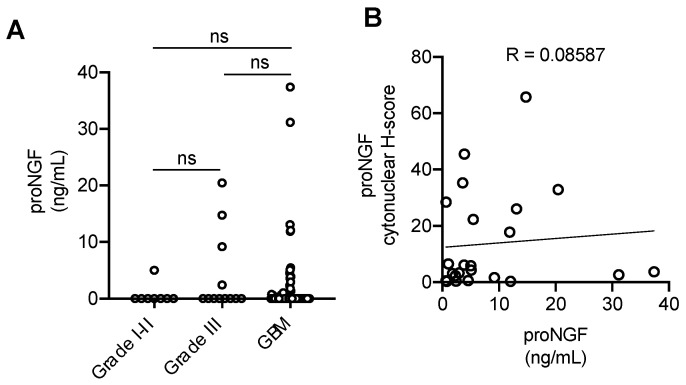
Plasma concentration of proNGF in GBM vs. low grade gliomas (**A**). ProNGF plasma quantifications were obtained by ELISA in plasma from grade I–II and grade III gliomas vs. GBM patients. ProNGF concentrations are represented in ng/mL. The mean proNGF concentration was 1.99 ng/mL (95% CI, 0.55–3.44) in GBM samples versus 3.90 ng/mL (95% CI, 0.56–8.36) in grade III and 0.6266 ng/mL (95% CI, 0–2.11) in grades I–II combined. Concentration distributions were compared using the Wilcoxon rank-sum (dichotomous) or Kruskal–Wallis (multiple comparisons) tests. Student’s *t*-test was used to evaluate the mean difference in plasma proNGF between grades I–II gliomas vs. GBM, and grade III gliomas vs. GBM. (**B**) Correlation analysis of patients positive for proNGF in plasma and IHC h-score.

ProNGF concentrations in plasma samples from GBM patients versus lower grade glioma patients are presented in Figure 2 and Table 2. ProNGF was detected in 19/72 (26%) of plasma samples from GBM patients, 4/12 (33%) from grade III patients, 0/6 (0%) from grade II patients and 1/2 (50%) from grade I patients (Table 2). The mean proNGF concentration was 1.99 ng/mL (95% CI, 0.55–3.44) in GBM samples versus 3.90 ng/mL (95% CI, 0.56–8.36) in grade III and 0.6266 ng/mL (95% CI, 0–2.11) in grades I–II combined. There was no significant difference in proNGF concentration between low grade gliomas and GBM (Figure 2A, Table 2). No significant correlation between plasma proNGF and proNGF h-scores was found in this cohort (Figure 2B).

Plasma concentration of proNGF was assayed by ELISA and categorized negative concentration (0 ng/mL), or as positive concentration (>0 ng/mL). A Chi-squared test was used to test statistical association. Statistically significant *p* values (*p* < 0.05) are shown in bold. ProNGF is the precursor for nerve growth factor.

### 2.3. Targeting proNGF Inhibits GBM Cell Growth In Vitro

**Figure 3:**
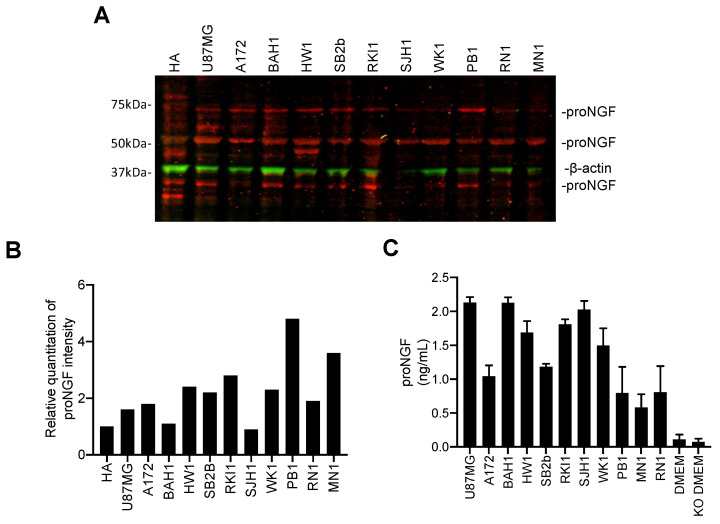
ProNGF protein expression in a panel of GBM cell lines. (**A**) Western blotting for proNGF was performed with cellular proteins extracted from HA (non-tumorigenic human astrocytes) cells and the following human GBM cell lines: U87MG, A172, BAH1, HW1, SB2b, RKI1, SJH1, WK1, PB1, RN1 and MN1. ProNGF was detected as 35, 55 and 75 kDa bands in all cell lines. (**B**) Densitometric analysis of the combined signal intensity obtained from the 3 proNGF bands. (**C**) ProNGF secretion was quantified by ELISA in conditioned medium of GBM cell lines. ProNGF concentrations are represented in ng/mL. Data shown are the mean ± SD of three independent experiments. DMEM: medium for U87MG and A172; KO DMEM: medium for all of the patient-derived GBM cells.

Western-blotting (Figure 3A) indicated that proNGF was expressed in all analyzed human GBM cell lines, including two standard GBM cell lines (U87MG and A172) and nine patient-derived GBM cell lines (BAH1, HW1, SB2b, RKI1, SJH1, WK1, PB1, RN1 and MN1). Human astrocytes (HA) were also included. Three major molecular weights (MW) for proNGF were observed at 35 kDa, 55 kDa and 75 kDa. Although the theoretical MW (based on amino acid sequence) of proNGF is ~26 kDa, proNGF can be extensively post-translationally modified, and in particular by glycosylation [15,21], and these proNGF apparent MW at 35, 55 and 75 kDa have already been reported by other groups using different antibodies [22,23,24]. Densitometric analysis of the combined signal intensity obtained from the three proNGF bands (Figure 3B) revealed that, with the exception of SJH1, all GBM cell lines exhibited an increased level of proNGF compared to the control HA cells. The secretion of proNGF was also detected in the conditioned medium of GBM cell lines (Figure 3C).

**Figure 4:**
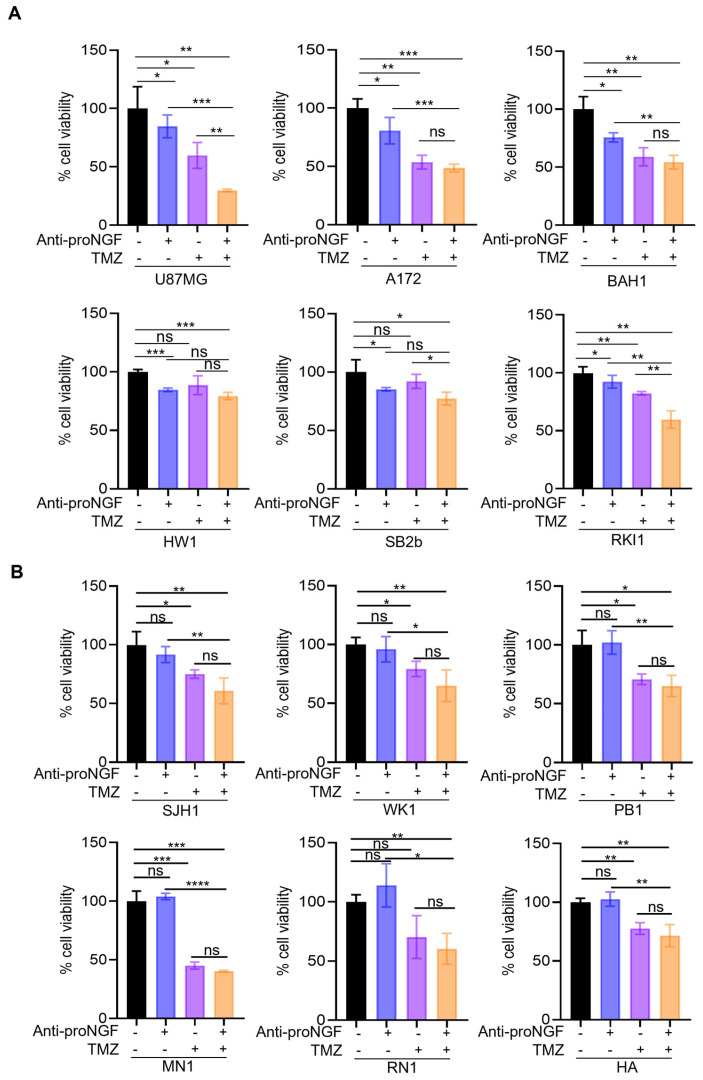
Biological effect of targeting proNGF in GBM cells. GBM cell growth was studied in a panel of GBM cell lines with methylated MGMT gene promoter (**A**) and unmethylated MGMT gene promoter (**B**) as well as human astrocytes (HA, served as a control cell line) treated with blocking antibodies against proNGF (anti-proNGF, 600 ng/mL), TMZ (50 μM) and anti-proNGF + TMZ for 72 h. Data shown are the mean ± SD of three independent experiments. * *p* < 0.05, ** *p* < 0.01, *** *p* < 0.001, **** *p* < 0.0001, Student’s *t*-test or ANOVA. proNGF: precursor for nerve growth factor, TMZ: Temozolomide. Black bars: PBS (vehicle control for anti-proNGF) + DMSO (vehicle control for TMZ); blue bars: anti-proNGF + DMSO (vehicle control for TMZ); purple bars: TMZ + PBS (vehicle control for anti-proNGF); orange bars: anti-proNGF + TMZ.

To investigate the functional features of proNGF in vitro, we first inhibited proNGF with anti-proNGF blocking antibodies in the same panel of GBM cell lines and the effect on both cell invasion and cell growth was analyzed. The anti-proNGF blocking antibody has no effect on GBM cell invasion (Supplementary Figure S1). In contrast, anti-proNGF tended to reduce cell growth in GBM cells with methylated MGMT gene promoter, however it did not affect cell growth in GBM cells with unmethylated MGMT gene promoter (Supplementary Figure S2A,B). Since MGMT promoter methylation has been reported to predict the outcome in GBM patients to alkylating chemotherapeutic agents [3], we then tested the effect of anti-proNGF in combination with TMZ on GBM cell viability. A further inhibition of cell viability by anti-proNGF was found in three (U87MG, RKI1 and SB2b) out of six GBM cell lines with methylated MGMT gene promoter when co-treated with TMZ compared to TMZ alone (Figure 4A). In contrast, anti-proNGF could not further sensitize cells to TMZ in GBM cell lines with unmethylated MGMT gene promoter (Figure 4B).

### 2.4. Targeting proNGF Inhibits GBM Tumor Growth In Vivo

**Figure 5:**
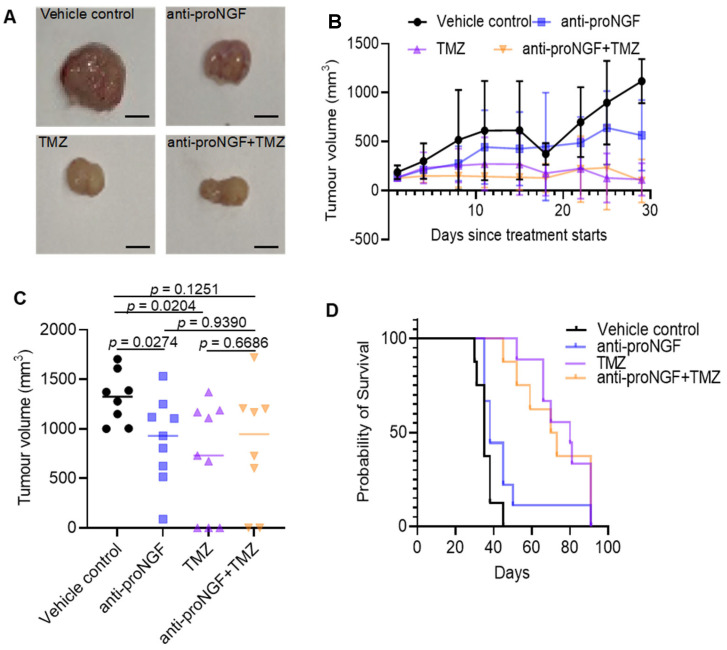
In vivo targeting of proNGF in a mouse model of GBM. A xenograft mouse model of GBM was used. The human U87MG cells were injected subcutaneously between the shoulder blades of BALB/c nude mice, along with blocking anti-proNGF antibody (12 μg, intratumoral injection, twice a week for 4 weeks) and/or TMZ (50 mg/kg body weight, IP daily for 5 days) treatment for a 4-week course. (**A**) Representative photographs of U87MG tumors excised from mice after 4 weeks of treatment. Scale bar, 5 mm. (**B**) The growth curves of U87MG tumors in BALB/c nude mice (*n* = 9 for each group). (**C**) Comparison of volume of U87MG tumors harvested from mice treated with blocking anti-proNGF antibody and/or TMZ for 4 weeks. (**D**) Kaplan-Meier survival curves of BALB/c nude mice treated with anti-proNGF antibody and/or TMZ. Vehicle control vs. anti-proNGF: *p* = 0.0639; Vehicle control vs. TMZ: *p* < 0.0001; Vehicle control vs. anti-proNGF + TMZ: *p* = 0.0003; Anti-proNGF vs. anti-proNGF + TMZ: *p* = 0.0160, TMZ vs. anti-proNGF + TMZ: *p* = 0.8515. Data shown are the mean ± SD of tumors with 9 mice per treatment group. ProNGF: precursor for nerve growth factor, TMZ: Temozolomide. Vehicle control: PBS (vehicle control for anti-proNGF) + 5%GMSO + 30% Polyethylene glycol 300 (PEG300) + double-distilled H_2_O (vehicle control for TMZ); anti-proNGF: anti-proNGF + 5%GMSO + 30% PEG300 + double-distilled H_2_O (vehicle control for TMZ); TMZ: TMZ + PBS (vehicle control for anti-proNGF); anti-proNGF + TMZ: anti-proNGF + TMZ.

To test the in vivo the impact of targeting proNGF on GBM tumor growth, we used a xenograft GBM mouse model in which human U87MG cells (MGMT methylated) were injected subcutaneously between the shoulder blades of BALB/c nude mice, along with anti-proNGF antibody alone or in combination with TMZ. In mice treated with antibody, anti-proNGF reduced the growth of U87MG tumors (*p* = 0.0274) (Figure 5A–C). However, anti-proNGF could not enhance TMZ-induced inhibition of tumor growth (*p* = 0.6686) (Figure 5A–C). Kaplan-Meier analysis showed that targeting proNGF tended toward producing a longer survival rate than vehicle control (*p* = 0.0639) (Figure 5D), but it failed to further prolong GBM mice survival as compared to TMZ treatment alone (*p* = 0.8515) (Figure 5D).

## 3. Discussion

This study reports, for the first time, the expression and biological effect of proNGF in GBM. The molecular pathogenesis of GBM remains to be elucidated and new molecular targets need to be identified in order to improve TMZ efficacy through the development of adjunct targeted therapies. Overall, the present data supports the potential value of proNGF as a clinical biomarker and therapeutic target in GBM.

In terms of gene expression, NGF mRNA abundance has not been reported to be linked to a particular clinicopathological parameter in GBM. Before investigating proNGF protein levels by immunohistochemistry, we have performed a data mining of NGF gene expression using GEPIA2 [25] and cBioportal [26] of GBM datasets in The Cancer Genome Atlas (TCGA) database [27] and GTEx [28]. Using GEPIA, no significant upregulation of NGF gene expression was found in GBM compared to normal brain tissues. Using cBioportal, an upregulation of NGF gene expression was detected in only 8% of GBM cases and no cases of NGF mRNA downregulation were found. Initial studies in yeast have suggested a correlation of about 50% between mRNA and protein levels [29], and in humans, global transcriptomic and proteomic analyses show that only an estimated 30% of changes in protein levels can be explained by corresponding variations in mRNA [30]. In this regard, biobanking GBM samples for protein analysis present more challenges than for investigating mRNA levels and this has recently been reviewed [31]. Interestingly, a proteogenomic investigation in colorectal cancer has also revealed that mRNA abundance does not reliably predict differences in tumoral protein levels [32]. This emphasizes the importance of directly analyzing proteins in cancer tissue, as performed in the present study, to identify potential new biomarkers and therapeutic targets in oncology; this is particularly true when studying a precursor protein form such as proNGF.

Increased proNGF levels have been reported in melanoma [16], breast [13], prostate [15] and thyroid [17] cancer tissues. We extend these observations by reporting here an increase in levels of proNGF in GBM and therefore, proNGF protein overexpression appears to be a feature of several cancers. Whether this increased expression could be of prognostic value to predict outcomes in GBM warrants further investigations in larger cohort of GBM with full clinical annotation. In addition, we also report for the first time that the level of proNGF, assessed by ELISA, is increased in the plasma of some GBM patients. While proNGF was undetected in most GBM plasma samples, a subgroup of GBM patients presented with a detectable level of proNGF. So far, one study has reported the presence of proNGF in the serum of some thyroid cancer patients [19] in concentrations comparable to what we report here in GBM. The presence of proNGF in the plasma/serum of cancer patients has not been investigated throughout the different types of human cancers and therefore it is unclear if what we report here is generally observed in cancer patients. Moreover, whether plasma proNGF originates from GBM remains to be determined, but the detection of proNGF in the plasma of some GBM patients raised the question of a potential value of proNGF as a blood biomarker for proNGF. There are currently no blood biomarkers for GBM, and this is limiting early diagnosis of the disease. The hypothesis that plasma proNGF could be used as a biomarker for GBM diagnosis is of interest but warrants further analysis of a large cohort of clinical plasma samples from GBM patients vs. non-GBM control samples.

The biological effect of proNGF appears to be different depending on physiological and pathological conditions. In the nervous system, proNGF has been described to be either neurotrophic or pro-apoptotic for neurons, depending on the presence and concentration of its membrane receptors [12]. In human tumors, there is a variety of biological activities associated with proNGF targeting, which is thought to be related to the expression and activities of its three different receptors, including the neurotrophin receptor p75^NTR^, the membrane protein sortilin and the tyrosine kinase receptor TrkA (11). In melanoma [16], breast [33] and thyroid cancer [34], proNGF blocking antibodies have been reported to have no impact on cancer cell growth but instead targeting proNGF led to a decreased cancer migration and invasion through a mechanism involving p75^NTR^ and sortilin [16]. In the present study, anti-proNGF antibodies were found to have no impact on GBM cell migration and invasion, but in contrast they decreased the growth of some GBM cells. This was observed both in vitro using cell cultures and in vivo using mouse subcutaneous xenograft tumors. The role of the different proNGF receptors in mediating proNGF effect is not clear at this stage because proNGF activity results from proNGF interaction with p75^NTR^, sortilin and TrkA, and therefore further mechanistic investigations are warranted. In addition, the secretion of proNGF in GBM conditioned medium was comparable regardless of the methylation status of MGMT promoter. This lack of proNGF neutralization effect on the growth of glioma cell lines without methylated MGMT promoter despite the presence of proNGF in the medium, could be related to the expression of proNGF receptors in glioma, emphasizing the need to clarify the expression of proNGF receptors in the future. Moreover, as proNGF is processed to give rise to NGF, it is not known if anti-proNGF antibodies have an effect on the production of NGF; this question has not been investigated in any other model and would require extensive biochemical investigations. Interestingly, in vitro, anti-proNGF was able to potentiate the efficacy of TMZ in some GBM cell lines, more particularly in methylated MGMT cell lines. However, in mouse subcutaneous xenograft tumors, although anti-proNGF slightly reduced tumor growth, it failed to potentiate the activity of TMZ, suggesting that targeting proNGF may have limited potential as adjunct therapy to TMZ. Nevertheless, it should be noted that further in vivo experiments, using different animal models, are needed before reaching any definitive conclusion. The subcutaneous tumor model that we have used here is interesting as a first approach and the results, although not showing a potentiation of TMZ efficacy by anti-proNGF at this stage, point to a reduced tumor growth after anti-proNGF treatment. In the future, orthotopic GBM models in which GBM cells will be injected directly in the brain need to be utilized. On that note, the need to use orthotopic GBM models is emphasized by the increasingly reported stimulatory impact of the neural environment in tumor progression [35]. Neuronal cells in the tumor microenvironment stimulate cancer cell growth and invasion by secreting neurotransmitters and other growth factors [35] and this is specially the case for GBM which are surrounded by neurons actively participating in cancer growth and invasion [36].

## 4. Materials and Methods

### 4.1. GBM Tissues and Plasma Cohort

This study was approved by the Human Research Ethics Committee of the University of Newcastle. Clinical and demographic information available for the cohort included age, gender, tumor grade and primary tumor site (Table 3). Formalin-fixed paraffin-embedded tumor tissue from maximal safe surgical resection was sourced from the Hunter Cancer Biobank (HCB) for seventy-two cases of glioblastoma, twelve cases of grade III glioma, six cases of grade II glioma, and two cases of grade I glioma. A block containing maximal tumor content was chosen from each patient and diagnosis of glioma grade was confirmed on H&E-stained sections by a neuropathologist. Matching plasma samples were also obtained at the time of diagnosis. Sample processing mirror standard diagnostic processing for plasma including a 15-min centrifugation at 1500 RPM followed by 10 min at 2500 RPM. Samples were processed immediately upon receipt into the laboratory and stored in −80 °C prior to retrieval.

### 4.2. Immunohistochemistry

Formalin-fixed paraffin-embedded tumor tissue was sliced into 4 µm full face sections and processed for 3′,3′-diaminobenzidine (DAB) immunohistochemistry using a Ventana Discovery Ultra (Roche, Indianapolis, IN, USA) by the HCB. Sections were labelled for anti-proNGF (0.8 µg/mL, catalogue number ANT005, Alomone labs, Jerusalem, Israel). As specified by the provider, this antibody specifically recognizes proNGF with no cross-reactivity with NGF as the target sequence of the antibody is specific for proNGF. All steps, from baking to chromogen addition were performed automatically by the instrument. Tissue sections were baked to slides and deparaffinized, and antigen retrieval then occurred at 95 °C/pH 9 with a total incubation time of 24 min prior to the addition of the primary antibody. The addition of the primary antibody was followed by a 32 min incubation at 36 °C. Slides were then incubated with secondary antibody (catalogue number MP-7401, Vector Laboratories, Newark, CA, USA) and revealed with DAB Peroxidase (HRP) Substrate Kit (catalogue number SK-4100, Vector Laboratories, Newark, CA, USA). Finally, slides were counterstained with Hematoxylin QS (catalogue number LS-J1045, Vector Laboratories, Newark, CA, USA), dehydrated and cleared in xylene before mounting in Ultramount No. 4 (catalogue number UM5-T, Hurst Scientific, Forrestdale, WA, Australia).

### 4.3. Digital Quantification of Immunohistochemistry

Following IHC staining, sample slides were digitized using the Aperio AT2 scanner (Leica Biosystems, Mount Waverley, VIC, Australia) at 40× absolute resolution. Quantitative IHC analyses were performed using the HALO^TM^ image analysis platform (version 3.3, Indica Labs, Albuquerque, NM, USA). Tissue classification algorithms were used to differentiate tissues and pixel intensity values corresponding to DAB staining were calculated using the Cytonuclear module [37], which detects and quantifies protein expression in the cytoplasm. Pixel intensity values were then used to determine the h-scores for each core (index calculated as the sum of 3 × % of pixels with strong staining + 2 × % pixels with intermediate staining + 1 × % pixels with weak staining). H-scores were analyzed as continuous variables, with summary statistics presented as group level medians and interquartile ranges (IQR). H-score distributions were compared using the Wilcoxon RankSum (dichotomous) or Kruskal Wallis (multiple comparisons) tests. To assess the primary hypothesis (difference in proNGF expression between pathological subtypes), a two-sided alpha of 0.05 was used. Statistical analyses were based on complete cases and performed using Prism (version 8.2.0, GraphPad Software, San Diego, CA, USA).

### 4.4. Enzyme-Linked Immunosorbent Assay (ELISA)

Plasma concentration of proNGF was determined using an ELISA kit (product number BEK-2226) from Biosensis Pty Ltd. (Thebarton, SA, Australia). The secretion of proNGF by GBM cells was also detected in the conditioned medium by using this ELISA kit. This sandwich assay allows for the quantification of proNGF using a sensitive and specific pre-coated plate with capture antibody, a biotinylated detection antibody and horseradish peroxidase-conjugated streptavidin. All kits were used as per the manufacturer’s instructions. Although all controls have been performed by the manufacturers, standard curve validation, spike recovery, linearity of dilution, intraplate reproducibility, limit of detection, and limit of quantitation were independently validated in our laboratory. All serum samples were tested 1:10 dilution. Plates were read with a Spectramax plate reader (Molecular Devices LLC, San Jose, CA, USA, M3). Optimization of the TMB incubation step was performed with every ELISA experiment. Plates were incubated in a box, and absorbance at 650 nm first read at the minimum incubation time specified in the kit protocol, and then every 5 min thereafter. TMB stop solution was added when the absorbance value (650 nm) of the highest concentration of the standard curve either reached 1.0 or above, or plateaued, and the rate of increase in absorbance was less than 0.3 every 5 min. Concentration distributions were compared using the Wilcoxon RankSum (dichotomous) or Kruskal Wallis (multiple comparisons) tests. To assess the primary hypothesis (difference in proNGF expression between pathological subtypes), a two-sided alpha of 0.05 was used. Statistical analyses were based on complete cases and performed using Prism (version 8.2.0, GraphPad Software, San Diego, CA, USA).

### 4.5. Cell Culture

Human glioblastoma cancer cell lines U87MG (HTB-14) and A172 (CRL-1620) were obtained from the American Type Culture Collection (ATCC, Manassas, VA, USA). Patient-derived glioblastoma cell lines BAH1, MN1, WK1, RN1, RKI1, HW1, PB1, SB2b, SJH1 were obtained from QIMR Berghofer Medical Research Institute (Dr Bryan Day, Brisbane, QLD, Australia). Human astrocyte (HA) cell line (catalogue number 1800) was obtained from ScienCell Research Laboratories (Wangara, WA, Australia). Details of GBM cell lines and patient-derived GBM cell lines together with their MGMT methylation status have previously been described [38,39]. U87MG and A172 cell lines were maintained in Dulbecco’s Modified Eagle’s Medium (Gibco, Thermo Fisher scientific, Waltham, MA, USA) with 10% Fetal Bovine Serum (JRH Biosciences, Macquarie Park, NSW, Australia), 5% penicillin- streptomycin (Gibco, Thermo Fisher scientific, MA, USA), 2 mmol/L _L_-glutamine (Gibco, Thermo Fisher scientific, MA, USA). Patient-derived cells were maintained in Knockout DMEM glutamine (KO DMEM, Gibco, Thermo Fisher scientific, Waltham, MA, USA) with 2% Stempro Neural Supplement (Gibco, Thermofisher scientific), 5% penicillin-streptomycin (Gibco, Thermo Fisher scientific, Waltham, MA, USA). HA cells were maintained in Astrocyte Medium from ScienCell Research Laboratories (catalogue number 1801), which contains basal medium, fetal bovine serum and astrocyte growth supplement and penicillin/streptomycin solution; they were also seeded on Poly-L-Lysine (catalogue number P4707, Sigma-Aldrich Pty Ltd., Macquarie Park, NSW, Australia) coated flasks. All cell cultures were kept in a humidified incubator at 37 °C with 5% CO_2_. Routine Mycoplasma testing was performed using the MycoAlert Mycoplasma Detection Kit (catalogue number LT07-118, Lonza, Basel, Switzerland). Cells were not maintained in culture for longer than 3 months to ensure passage number remained fit for purpose.

### 4.6. Preparation of Conditioned Medium

GBM cells (4 × 10^5^ cells) were seeded in a six-well plate. Twenty-four hours later, cells were washed three times with PBS and then incubated in DMEM (for U87MG and A172) or KO DMEM (for patient-derived GBM cells) for another forty-eight hours. Conditioned medium was centrifuged for 10 min at 2000 rpm and then filtered through a 0.22 μm filter (Merck, Bayswater, VIC, Australia) to remove the contaminating cells or cell debris.

### 4.7. Protein Extraction and Western Blotting

Protein extraction from cell lines and Western blotting experiments were performed as previously described [15]. Anti-proNGF antibody (catalogue number M-1738, Biosensis, Thebarton, SA, Australia) was used at a dilution of 1:500 and a β-actin antibody (catalogue number A1978, Sigma-Aldrich) was used at a 1:5000 dilution as the equal loading control. As specified by the provider, the proNGF antibody is specific of proNGF as the target sequence is (C-HTIPQAHWTKLQ, aa: 30–41) of human proNGF protein which is located on the pro-domain of the proNGF full-length protein (not present in NGF).

### 4.8. In Vitro Cell Growth and Invasion Assays

The cell growth assay was carried out using Cell Titer-Blue^®^ (Promega, Hawthorne, VIC, Australia) according to the manufacturer’s instructions. Cells were plated at 5000 cells per well in a Corning Costar 96-well plate (catalogue number CLS3599, Sigma-Aldrich Pty Ltd., Macquarie Park, NSW, Australia). Cells were then treated with anti-proNGF (600 ng/mL, catalogue number M-1738, Biosensis, Thebarton, South Australia; vehicle control: PBS), TMZ (50 μM, catalogue number S1237, Selleck Chem, Sapphire Bioscience, NSW, Australia; vehicle control: DMSO) and anti-proNGF + TMZ for 72 h. At the end of treatment, Cell Titer-Blue^®^ reagent was added into each well followed with detection of fluorescent signal by Fluostar Optima (BMG Labtech, Ortenberg, Germany) with a 560/590 nm filter set.

The invasion assay was carried out using the 6.5 mm Transwell^®^ 8.0 µm Pore Polycarbonate Membrane Insert (Corning^®^, Sigma-Aldrich). To form the invasion barrier, Matrigel (BD Bioscience, Piscataway, NJ, USA) was diluted 1:8 with serum-free DMEM. The membrane was rehydrated with pre-warmed serum-free media for 30 min at 37 °C. 4 × 10^4^ cells in serum-free media were seeded into the upper invasion chamber while the bottom chamber had media with serum added. Cells were treated with 600 ng/mL anti-proNGF (600 ng/mL, catalogue number M-1738, Biosensis, Thebarton, SA, Australia). An isotope antibody was used as an antibody control (catalogue number #M-1763-FT, Biosensis Pty Ltd., Thebarton, SA, Australia) and was used at the same concentration. After 48 h, non-invaded cells in the upper chamber were gently rinsed away with phosphate-buffered saline (PBS) twice. For quantitation of invasion assay, cell numbers were counted in five squares.

### 4.9. In Vivo Experiments in Murine Xenograft Model

The human GBM U87MG cells (5 × 10^6^ cells per mouse) were injected subcutaneously between shoulder blades of female BALB/c nude mice (8- to 10-weeks-old). When the smallest tumor reached a size of 100 mm^3^, the mice were randomly divided into groups (9 mice per group) followed by treatment with anti-proNGF (12 µg, intratumoral injection, twice a week for 4 weeks; vehicle control: PBS) and/or TMZ (catalogue number S1237, Selleck Chem, Sapphire Bioscience, Redfern, NSW, Australia) (50 mg/kg body weight, IP daily for 5 days; vehicle control: 5%GMSO + 30% Polyethylene glycol 300 (PEG300) + double-distilled H_2_O) treatment for a 4-week course. At the end of the 28-day treatment, the experiment continued for up to 100 days post-inoculation to allow for significant improvement in the mice treated with the drugs unless the tumor volume reached 1000 mm^3^. Mice were then euthanized, and tumor tissues were collected, weighed, and processed for histological examination. Experimental sample size calculation was used to estimate the sample size, which represents an optimum number to attain statistical significance of *p* < 0.05 with a 90% probability. This study was approved by the Animal Research Ethic Committee of Sydney University and all experiments were performed in accordance with relevant guidelines and regulations.

### 4.10. Statistical Analysis

Statistical analysis was performed using GraphPad Prism (La Jolla, CA, USA). Single comparisons were performed using unpaired two-sided Student’s *t*-test. Multiple comparisons were performed using one-way analysis of variance (ANOVA) or two-way analysis of variance with Dunnett’s or Tukey’s correction. The non-parametric Kruskal-Wallis test was performed where data was not normally distributed. Correlation was examined with Pearson’s correlation. A *p*-value less than 0.05 was considered statistically significant. All in vitro and in vivo experiments were repeated at least three times. All materials used and results generated from the study were included for statistical analysis. No exclusion of data points was used. All data generated or analyzed during this study is included in this published article. Data are presented as mean ± standard deviation (SD).

## 5. Conclusions

In conclusion, this exploratory study demonstrated an increased level of proNGF in GBM tissues, proNGF presence in the blood of some GBM patients, and its potential involvement in GBM growth, suggesting that this growth factor is a potential new clinical biomarker and deserves further exploration as a potential therapeutic target. Further investigations of GBM larger cohorts are warranted to assess the clinical relevance of proNGF as a clinical biomarker and its value as a therapeutic target should be further investigated in various in vivo models.

## Figures and Tables

**Table 1:** Association between proNGF expression (staining intensity, h-score) and clinicopathological parameters in gliomas.

Parameter	proNGF Intensity		*p*-Value
	Low	High	
Sex			0.2927
Female	17 (46%)	20 (54%)	
Male	29 (54%)	25 (46%)	
Age			0.9892
≤63	24 (51%)	23 (49%)	
>63	22 (50%)	22 (50%)	
Grade			
I–II	7 (87.5%)	1 (12.5%)	<0.0001
III	5 (42%)	7 (58%)	
GBM	34 (48%)	37 (52%)	
Tumor site			0.0848
Frontal	19 (49%)	20 (51%)	
Temporal	19 (66%)	10 (34%)	
Other	7 (30%)	16 (70%)	

**Table 2:** Association between proNGF concentration in plasma and clinicopathological parameters in glioma.

Parameter	proNGF		*p*-Value
	Negative	Positive	
Sex			0.1562
Female	30 (81%)	7 (19%)	
Male	38 (69%)	17 (31%	
Age			0.9151
≤63	37 (79%)	10 (21%)	
>63	31 (69%)	14 (31%)	
Grade			0.3415
I–II	7 (87.5%)	1 (12.5%)	
III	8 (67%)	4 (33%)	
GBM	53 (74%)	19 (26%)	
Tumor site			0.6417
Frontal	28 (72%)	11 (28%)	
Temporal	23 (77%)	7 (23%)	
Other	17 (55%)	6 (45%)	

**Table 3:** Demographics and clinical characteristics of patient cohort.

Characteristic	Subgroup	Total
Participants	N	92
Sex	Female	37 (40%)
	Male	55 (60%)
Age at diagnosis	Median (min, max)	63 (17, 82)
	Median (Q1, Q3)	63 (56.5, 72)
Grade	I	2 (2.2%)
	II	6 (6.5%)
	III	12 (13%)
	GBM	72 (78%)
Tumor site	Frontal	39 (42%)
	Temporal	30 (33%)
	Parietal	15 (16%)
	Other	8 (9%)

## Data Availability

The data presented in this study are available on request from the corresponding author.

## Supplementary Materials

The following supporting information can be downloaded at: https://www.mdpi.com/article/10.3390/ijms24021616/s1.
