# Supplementary material for: ProNGF Expression and Targeting in Glioblastoma Multiforme
Source: Int J Mol Sci. 2023 Jan 13;24(2):1616. doi: 10.3390/ijms24021616 (PMC9863529; doi:10.3390/ijms24021616)
Supplement: Supplementary file 1 [file ijms-24-01616-s001.zip › ijms-2062991-supplementary.pptx]

## Slide 1
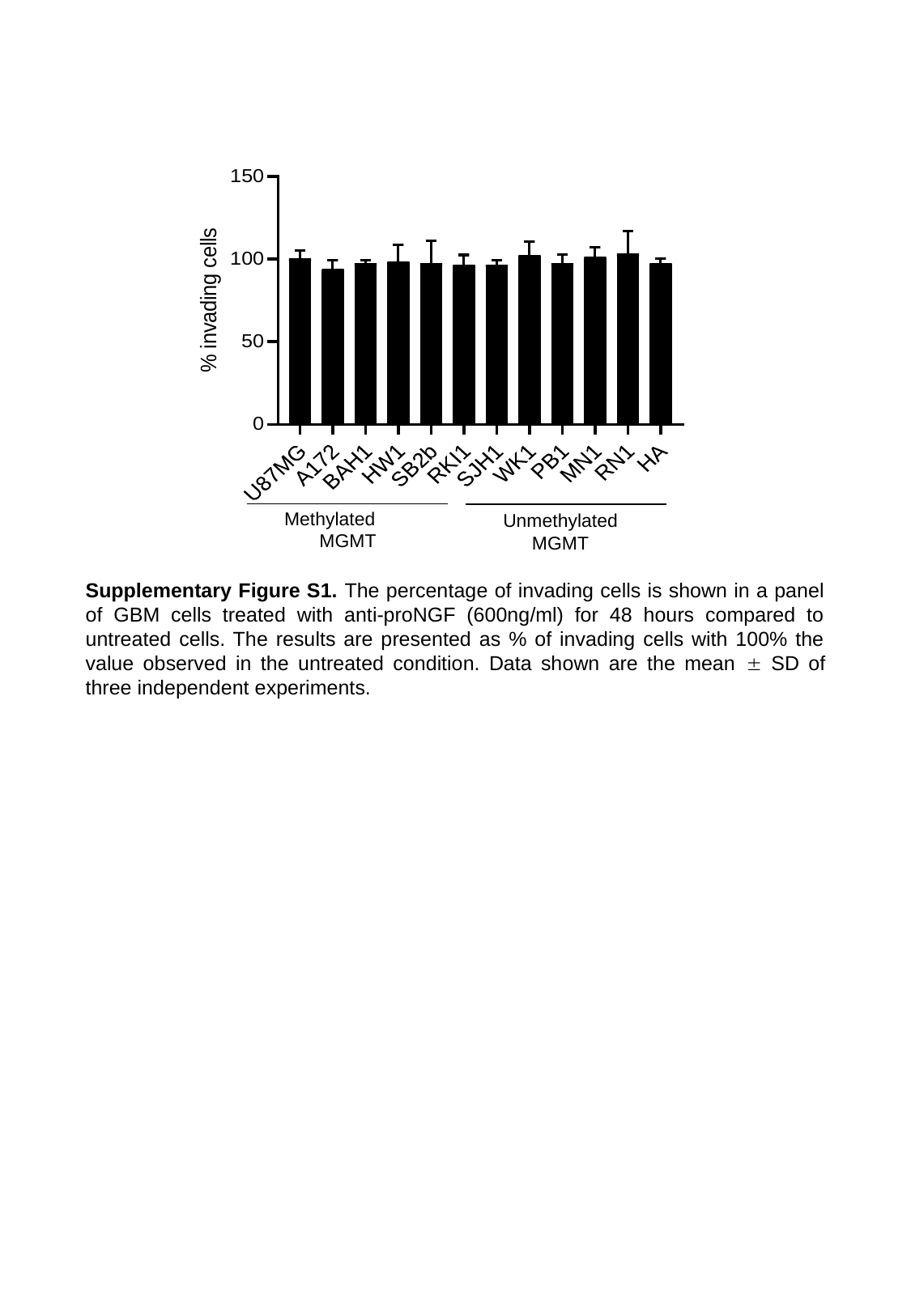

Methylated
MGMT
Unmethylated
MGMT
Supplementary Figure S1. The percentage of invading cells is shown in a panel of GBM cells treated with anti-proNGF (600ng/ml) for 48 hours compared to untreated cells. The results are presented as % of invading cells with 100% the value observed in the untreated condition. Data shown are the mean ± SD of three independent experiments.

## Slide 2
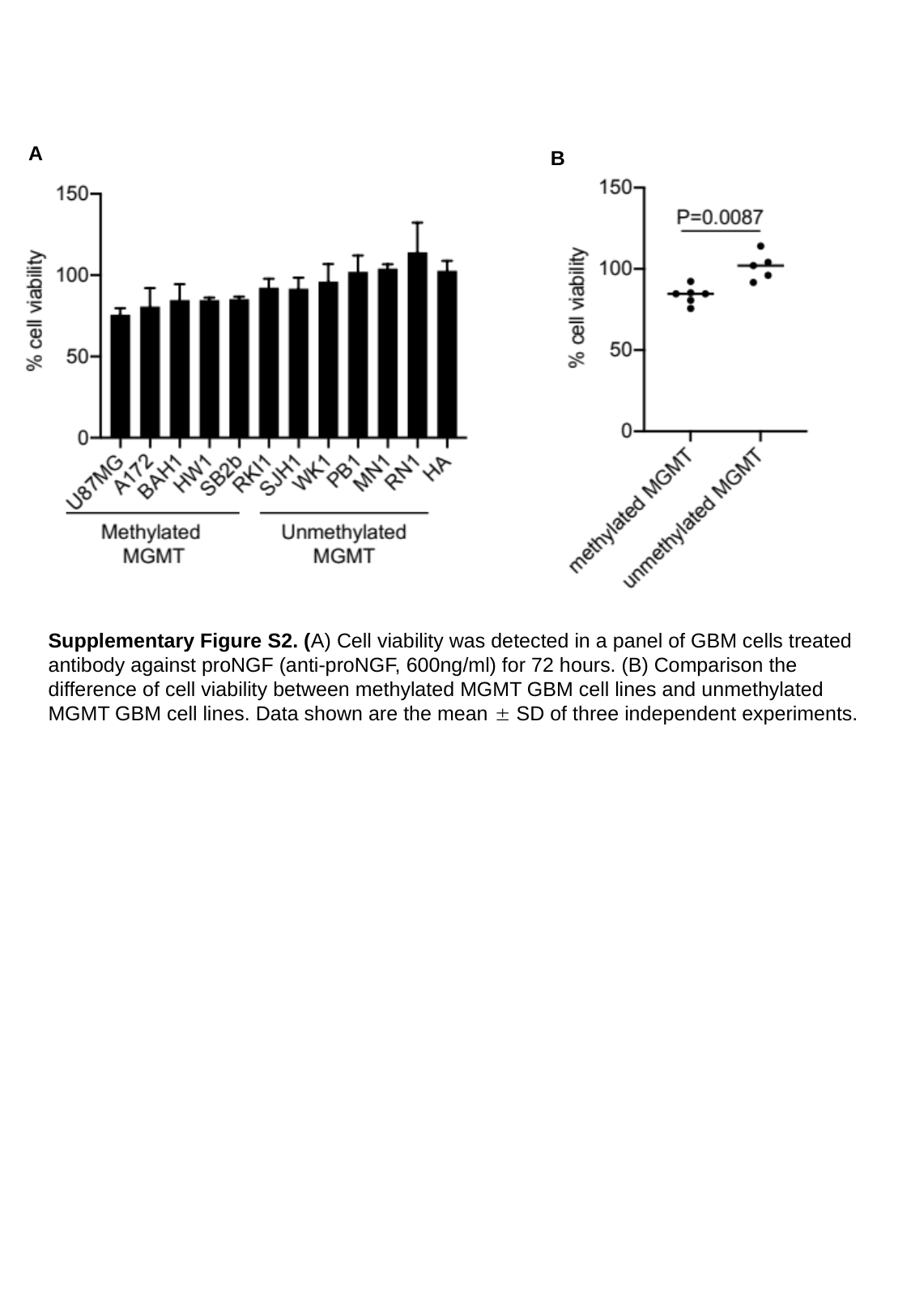

A
B
Supplementary Figure S2. (A) Cell viability was detected in a panel of GBM cells treated antibody against proNGF (anti-proNGF, 600ng/ml) for 72 hours. (B) Comparison the difference of cell viability between methylated MGMT GBM cell lines and unmethylated MGMT GBM cell lines. Data shown are the mean ± SD of three independent experiments.
